# Cryopreservation of Rubus viruses in raspberry shoot tips via droplet-vitrification: assessment of viral preservation, localization, and post-thaw transmission capacity

**DOI:** 10.1186/s13007-025-01454-w

**Published:** 2025-10-27

**Authors:** Xiao-Yan Ma, Dag‑Ragnar Blystad, Qiao-Chun Wang, Zhibo Hamborg

**Affiliations:** 1 Division of Biotechnology and Plant Health, Norwegian Institute of Bioeconomy Research, Ås, 1433 Norway; 2 College of Horticulture, Northwest A&F University, Yangling, 712100 Shaanxi China

**Keywords:** Aphid transmission, Cryopreservation, Droplet-vitrification, In situ hybridization, Micrografting, Raspberry, Rubus viruses, Shoot tip

## Abstract

We report the successful cryopreservation of three economically important Rubus viruses: raspberry bushy dwarf virus (RBDV), black raspberry necrosis virus (BRNV), and Rubus yellow net virus (RYNV), using shoot tip cryopreservation in four raspberry cultivars. Virus-infected shoot tips (approximately 1.0 mm in length) containing 3–4 leaf primordia (LPs) were cryopreserved using the droplet-vitrification technique. In the cultivars ‘Zlatá Královna (ZK)’ and ‘Tulameen (TUM)’, over 90% of shoot tips survived, and more than 90% regenerated into whole shoots. All three viruses were successfully preserved in the cryopreserved tissues, with recovery rates varying depending on virus type and cultivar: RBDV was recovered at rates of 86% in ‘ZK’ and 87% in ‘TUM’; BRNV at 66% in ‘ZK’ and 45% in ‘TUM’; and RYNV at 96%, 94%, and 86% in ‘Fairview’, ‘Stiora’, and ‘ZK’, respectively. To investigate viral localization in shoot tips, in situ hybridization was used. RBDV and RYNV infected a broad range of meristematic tissues, including the apical dome and LPs, whereas BRNV showed a more limited distribution. Virus distribution varied not only among virus species but also across raspberry cultivars, suggesting genotype-specific patterns of virus localization. Post-cryopreservation viral activity was verified using micrografting and aphid transmission assays. RBDV, BRNV, and RYNV were all successfully transmitted to healthy plants via micrografting, indicating the preservation of viral infectivity. Furthermore, BRNV was effectively transmitted by large raspberry aphids from cryopreserved materials, confirming vector-mediated transmission capacity post-thaw. Overall, this study demonstrates that shoot tip cryopreservation via droplet-vitrification is a reliable and effective strategy for preservation of biologically active Rubus viruses. This approach offers a valuable biotechnological tool for virus maintenance in support of diagnostic, breeding, and virology research.

## Introduction

Plant viral diseases pose a significant challenge to the sustainable cultivation, propagation, and fruit production of raspberry plants [1–3]. Unlike fungal and bacterial infections, which can often be controlled with chemical treatments, viral infections are difficult to manage once established [4, 5]. Compound infections, in particular, contribute significantly to production losses. For example, raspberry mosaic disease results from combinations of aphid-transmitted viruses, including black raspberry necrosis virus (BRNV, *Sadwavirus rubi*), raspberry leaf mottle virus (RLMV, *Closterovirus macularubi*), and Rubus yellow net virus (RYNV, *Badnavirus reterubi*) [2, 6, 7]. Similarly, the raspberry crumbly fruit complex arises from interactions between pollen-transmitted raspberry bushy dwarf virus (RBDV, *Idaeovirus rubi*) and aphid-transmitted viruses such as RLMV, RYNV, and raspberry latent virus (RpLV, *Ilarvirus TSV*) [2, 7, 8]. Therefore, research on these viruses is essential for ensuring the health and sustainability of the raspberry industry.

Preserving viruses that infect raspberry plants is crucial for research and practical applications, such as antibody production and providing positive controls in virus detection assays [9–11]. However, since plant viruses are intracellular obligate parasites that require living host tissue for replication, they cannot survive outside their hosts, making long-term preservation challenging.

Cryopreservation, which involves storing biological materials at ultra-low temperatures, has emerged as an effective method for the long-term preservation of plant viruses [12–16]. By preserving infected plant tissues or cells in liquid nitrogen (LN), cryopreservation maintains the viability of both the host tissue and the virus. This approach eliminates the need for continuous culture, which can lead to genetic instability and loss of viral infectivity [17, 18]. Cryopreservation has been successfully applied to preserve plant viruses and viroids infecting crops such as potatoes [19], apples [20, 21], strawberry [16], and panax ginseng [22].

In contrast, conventional virus preservation methods are largely based on storing purified virus particles or infected plant sap at low temperatures (e.g., − 20 °C–− 80 °C), or by freeze-drying [23]. While these techniques can be useful for short-term storage and specific diagnostic applications, they often fail to maintain viral infectivity over extended periods—especially for viruses that are unstable outside the host or sensitive to drying [19, 24]. Furthermore, many important raspberry viruses cannot be mechanically transmitted, which limits the utility of purified particles for re-infection or maintenance [25]. Therefore, traditional preservation does not support biological functionality or host-virus interactions, which are crucial for studies on transmission, symptom development, and host response. Cryopreservation of virus-infected plant tissues offers a more biologically relevant alternative that preserves viruses in their infectious form within living host cells.

Various cryopreservation techniques have been developed for raspberry plants, including encapsulation–dehydration [26], encapsulation–vitrification [27], vitrification [26], and droplet-vitrification [28]. Among these methods, droplet-vitrification is the most widely used due to its simplicity and effectiveness in preserving plant viruses [16, 20, 22, 29] and has been shown to successfully preserve viruses within plant tissue, maintaining their viability and infectivity after thawing [20, 29].

Despite its effectiveness, droplet-vitrification faces challenges due to the uneven distribution of viruses within plants. Virus concentrations often decrease toward the growing tip of the plant, known as the apical dome (AD), and the uppermost sections may have reduced viral loads or even be virus-free [30]. During shoot tip cryopreservation, only the cells in the outermost layers of the AD and the youngest developing leaves, called leaf primordia (LPs), typically survive freezing in LN [31–33]. Consequently, plants regenerated from these shoot tips may lack the virus, hindering efforts to preserve it [34, 35]. Nevertheless, successful preservation of viruses that infect AD, such as apple stem grooving virus (ASGV, *Capillovirus mali*) [20], has been achieved. Understanding the distribution of viruses within plant tissues is therefore crucial for improving cryopreservation strategies aimed at both virus eradication and preservation. By selecting appropriate tissue samples that contain the virus and optimizing cryopreservation protocols, we can enhance the efficiency of virus preservation.

Viruses can lose activity and transmission ability after preservation due to potential damage during storage processes. Freezing and thawing cycles can adversely affect viral particles, protein function, or the viability of host tissues, leading to a loss of infectivity [36–39]. Wang et al. (2018) reported a 0.13% change in preserved viral nucleotide sequences following cryopreservation, but this did not affect its viability [20]. While studies have confirmed the stability of viral genetic sequences post-cryopreservation [19, 20], there is a lack of reports verifying the infectivity of these viruses through biological methods such as grafting and insect vector transmission.

Based on these considerations, the objectives of this study were to: (1) investigate the distribution of BRNV, RBDV and RYNV in raspberry shoot tips using in situ hybridization (ISH); (2) evaluate the efficiency of droplet-vitrification cryopreservation for preserving these viruses in infected raspberry cultivars; and (3) verify the post-cryopreservation viability of the viruses through micrografting and aphid transmission assays.

## Materials and methods

### Plant materials

Virus-infected raspberry cultivars (*Rubus idaeus*) were selected for cryopreservation and viability study of the virus’s post-cryopreservation: ‘Zlatá Královna (ZK)’, co-infected with RBDV, BRNV and RYNV; ‘Tulameen (TUM)’, co-infected with RBDV and BRNV; ‘Fairview (FV)’ and ‘Stiora’, both infected with RYNV. In addition, healthy plants of ‘Ninni’ were used in the micrografting experiment. All in vitro shoots of the four virus-infected and healthy raspberry cultivars were cultured on a shoot maintenance medium (SMM), composed of Murashige and Skoog (MS) medium [40] supplemented with 30 g/L sucrose, 0.5 mg/L 6-benzylaminopurine (BAP), 0.1 mg/L indole-3-butyric acid (IBA), 36 mg/L Ethylenediamine di-2-hydroxyphenyl acetate ferric (Fe-EDDHA), and 6 g/L agar. The pH was adjusted to 5.7 prior to autoclaving at 121 ℃ for 20 min. Cultures were maintained at 22 ± 2 ℃ under a 16-h photoperiod with cool-white fluorescent light at an intensity of 50 µmol m⁻² s⁻¹. Subculturing was performed every 4 weeks. In addition, Raspberry cultivar ‘Lloyd George’ (LG), ‘Bliss’, and ‘Malling Jewel (MJ)’ were grown in a greenhouse at 24 ± 2℃ with a 16-h photoperiod and a light intensity of 200 µmol m⁻² s⁻¹, were involved in this study as virus positive controls and virus localization study of RYNV, RBDV and BRNV respectively.

### Virus detection

Virusstatus was assessed by reverse transcription-polymerase chain reaction (RT-PCR) on three occasions. The first assessment was conducted on infected in vitro shoots prior to cryopreservation experiments to confirm that all in vitro shoots used were virus infected. The second assessment was carried out on shoots regenerated from cryopreserved shoot tips after 3 months of shoot regeneration. The third assessment was conducted 2 months post-aphid inoculation and 2 weeks after micrografting.

Total RNA was extracted from tissue (0.5 g fresh weight) using Norgen Plant/Fungi RNA Kit (Norgen Biotek Corp, Thorold, ON, Canada). The aphid samples, collected in the DNA/RNA Shield solution (Zymo Research, CA, USA), were subjected to RNA extraction following the protocol described by Sapkota et al. [3], using the Direct-zol RNA Miniprep Kit (Zymo Research, CA, USA), according to the manufacturer’s instructions. The total RNA quality was assessed using a NanoDrop 1000b spectrophotometer (NanoDrop Technologies, Wilmington, DE, USA), and then the RNA was reverse transcribed to cDNA using SuperScript^®^ IV reverse transcriptase (Thermo Fisher Scientific, Germany) following the manufacturer’s instruction. PCR amplification was performed on the cDNA using specific primers (Table 1). Each 25 µL PCR reaction mixture contained 2.5 µL buffer, 0.5 µL dNTPs, 1 µL each primer at 10 µM concentration, 2 µL cDNA, 0.2 µL *Taq* DNA polymerase (5 U/ µL) (Thermo Fisher Scientific, Germany), and 17.8 µL RNase-free water. The PCR reaction conditions were as follows: an initial denaturation at 94 °C for 5 min; 35 cycles of 94 °C for 30 s, 56 °C for 30 s, and 72 °C for 50 s; followed by a final extension at 72 °C for 10 min. The reactions were then held at 4 °C. The PCR products were separated electrophoresis in 1.5% agarose gel and stained with SYBR safe DNA stain (Invitrogen, Thermo Fisher Scientific, USA).

**Table 1:** Primers used for reverse transcription polymerase chain reaction (RT-PCR) and in situ hybridization (ISH)

Primers used	Primer sequence (5’-3’)	Size (bp)	References
RBDV-MPF	GGGTTAAAGCTGCCTTCTCTTT	305	This study
RBDV-MPR	CTCACCAGTACCGTTTTGAACA		
BRNV-F3	CTGCCGCACTTATAAAGAGT	331	This study
BRNV-R3	CAGATGACCTGCCATAATAT		
RYNV_F_alternative	TCCAAAACCTCCCAGACCTMAAAC	350	This study
RYNV_R_alternative	TTGTTATATAATCACAAAAAGCTAACCAC		
LCO1490	GGTCAACAAATCATAAAGATATTGG	700	[46]
HCO2198	TAAACTTCAGGGTGACCAAAAAATCA		
ISH-T7-RBDV (sense)	TAATACGACTCACTATAGGA**GGGTTAAAGCTGCCTTCTCTTT**	305	This study
ISH-T3-RBDV (antisense)	AATTAACCCTCACTAAAGGG**CTCACCAGTACCGTTTTGAACA**		
ISH-T3-BRNV (sense)	AATTAACCCTCACTAAAGGG**CTGCCGCACTTATAAAGAGT**	331	This study
ISH-T7-BRNV (antisense)	TAATACGACTCACTATAGGG**CAGATGACCTGCCATAATAT**		
ISH-T3-RYNV (sense)	AATTAACCCTCACTAAAGGG**TCCAAAACCTCCCAGACCTMAAAC**	350	This study
ISH-T7-RYNV (antisense)	TAATACGACTCACTATAGGG**TTGTTATATAATCACAAAAAGCTAACCAC**		

‘RBDV’= raspberry bushy dwarf virus; ‘BRNV’=black raspberry necrosis virus; ‘RYNV’=Rubus yellow net virus. The bold parts are virus sequences and the unbold parts are transcription promoters in ISH primers

### Virus localization by in situ hybridization (ISH)

Shoot tips (about 2 mm in size) containing 4–5 LPs and shoot segments were used for ISH. The samples were fixed in fixative FAA (formaldehyde, alcohol, acetic acid) for 24 h, dehydrated through an ethanol series and embedded in paraffin, according to Lee et al. [41]. To generate DNA templates for probe synthesis, specific regions of the RBDV (305 bp), BRNV (331 bp), and RYNV (350 bp) genomes were amplified using RT-PCR with primers (Table 1). Digoxigenin (DIG)-11-UTP probes were synthesized directly from PCR products using T3 or T7 RNA polymerase (DIG RNA Labelling Kit (T7/T3), Roche, Germany) to produce sense and antisense probes. Section (8 μm thick) were prepared using a Rotary Microtome (Leica RM2255, Germany) and collected on Adhesion microscope slide glass (Thermo Fisher Scientific, Germany). ISH was performed as described by Zhang et al. [42], with minor modifications. Briefly, the tissue sections were rehydrated through a graded ethanol series and treated with 1 µg/ml proteinase K (Thermo Fisher Scientific, Germany) to remove proteins. The sections were then incubated in 0.1 M triethanolamine for 5 min. Acetic anhydride was added to the triethanolamine solution at 400 µL per 100 ml, and the incubation continued for another 10 min, following by series ethanol dehydration. After dehydration, DIG-labeled RNA probes were added to the hybridization solution from DIG Northern Starter Kit (Roche, Germany) and incubated at 68℃ overnight in a wet box. The sections were then washed in a buffer consisting of 2x saline-sodium citrate (SSC) buffer and 0.1x SSC buffer for 10 min and 30 min at 50℃, respectively. Then the sections were washed twice in Maleic acid buffer (0.15 M NaCl and 0.1 M Maleic acid, pH 7.5) for 15 min at room temperature. After washing, the sections were placed in blocking solution (Roche, Germany) for 1 h, followed by incubation for 1 h at RT with anti-DIG-Alkaline Phosphatase (AP) (1:3000 dilution) in blocking solution. The sections were subsequently washed four times in washing buffer (0.15 M NaCl, 0.1 M Maleic acid, and 0.3% (v/v) Tween 20) at room temperature, with each wash lasting 10 min. Preincubation in AP buffer (0.1 Tris-HCl, pH 9.5, and 0.1 M NaCl) was performed for 10 min. The colour reaction was performed using the substrate solution (NBT/BCIP; Promega, USA) in the dark. The colour reaction was stopped using stop solution (10 mM Tris-HCl, pH 8, and 1 mM EDTA). Results were examined with a light microscope (Leica, Germany).

### Cryopreservation

Droplet-vitrification cryopreservation was applied to cryopreserve virus, according to Ma et al. [28]. Shoot tips (1.0 mm in size) with 3–4 LPs were excised from the virus-infected shoots and stepwise precultured on MS medium (6 g/L agar, pH 5.7) containing 0.25 M, 0.5 M and 0.75 M sucrose, with each step lasting for 1 day. Precultured shoot tips were treated with loading solution composed of 2 M glycerol and 0.4 M sucrose in MS medium at pH 5.7 at room temperature for 20 min, followed by treatment of plant vitrification solution 2 (PVS2) at room temperature for 20 min. PVS2 contains 15% (w/v) ethylene glycol, 15% (w/v) dimethyl sulfoxide (DMSO), 30% (w/v) glycerol and 0.4 M sucrose in MS medium at pH 5.7 [43, 44]. Following PVS2 vitrification, PVS2 droplets (3 µL), each containing one shoot tip, were placed onto aluminum foil (3 × 0.8 cm) and directly immersed in LN for 2 min. After then, the aluminum foils were thawed by rapid transferring into an unloading solution (0.75 M sucrose in MS medium at pH 5.7) at room temperature for 20 min. The cryopreserved shoot tips were post-cultured on MS medium (6 g/L agar, pH 5.7) containing 0.75 M sucrose in the dark for 3 days and then transferred to SMM medium for shoot regeneration at culture conditions as used for maintenance of the infected in vitro shoots. Subculturing was performed once every 4 weeks. In order to select an optimal size of shoot tips for virus cryopreservation, an additional experiment was conducted, in which three different sizes of shoot tips, i.e., 0.5 mm with 1–2 LPs, 1 mm with 3–4 LPs, and 2 mm with 5–6 LPs were used for virus cryopreservation.

### Virus transmission by micrografting

For virus transmission by micrografting, four-week-old in vitro stock shoots were used. To assess the activity of RBDV and BRNV, shoots (1–1.5 cm in length) with two well-developed leaves were excised from the top parts of the regenerated virus-infected ‘ZK’ after cryopreservation. These shoots were used as scions and grafted onto RBDV and BRNV-free ‘Stiora’ shoot segments. For testing the activity of RYNV, similar sized shoot segments were excised from RYNV-infected shoots of ‘ZK’ and used as rootstocks, while virus-free ‘Ninni’ shoots served as scions. Micrografting was performed as described by Hao et al. [45]. A ‘V’-shaped cut (~ 0.5 cm in length) was made at the base of the scions, and a vertical cut of the same length was made at the top of the rootstocks. The ‘V’-shaped scions were then inserted into the vertical cut of the rootstocks to complete the grafting process. The micrografted plants were cultured on SMM and grown under the same culture conditions as described for the plant material for 2 weeks. Samples from the micrografted plantes were collected at 0, 3, 7, and 14 days post-micrografting for ISH analysis.

### Virus transmission by aphid

**Fig. 1:**
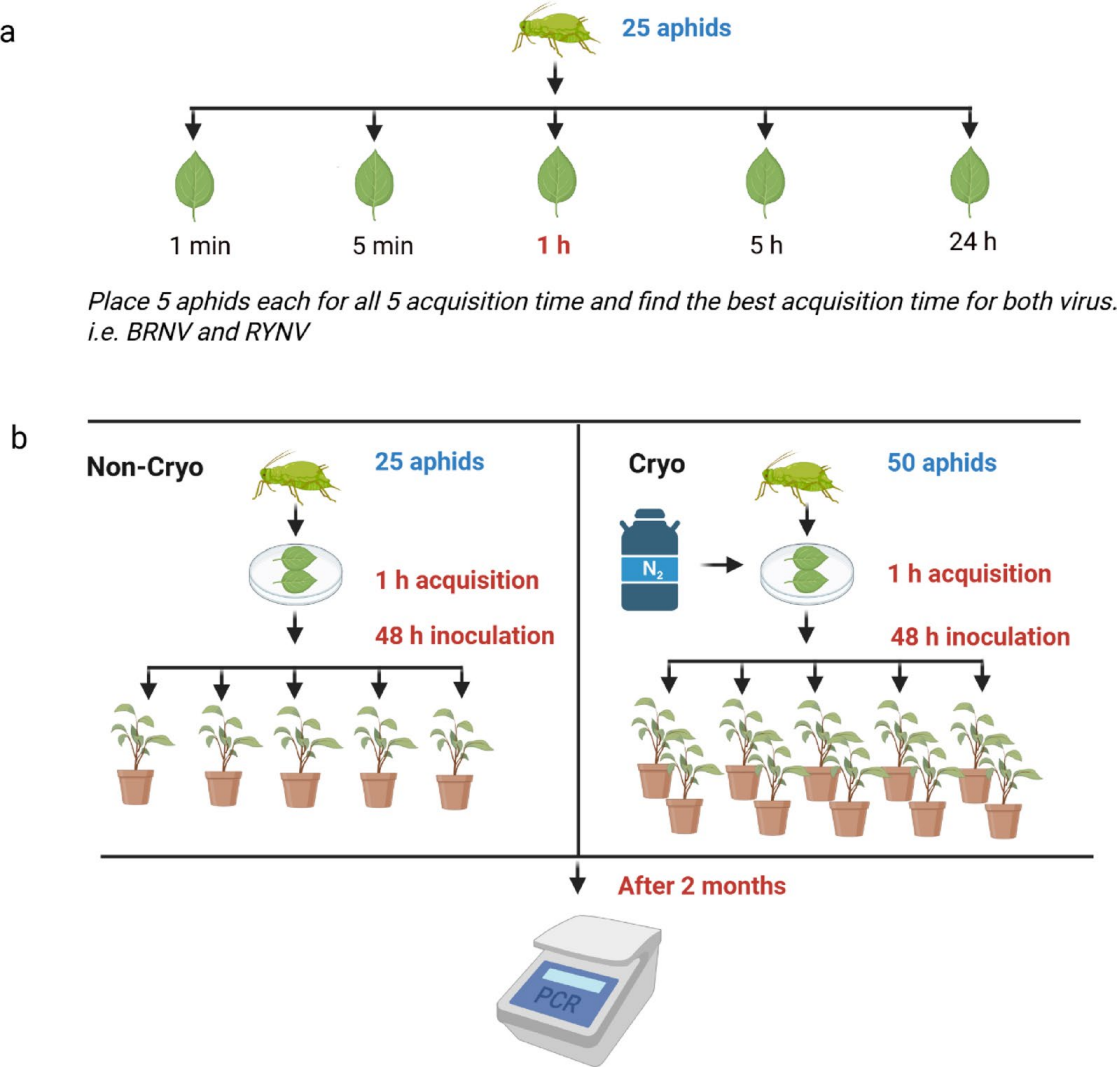
Schematic illustration of aphid-mediated transmission of cryopreserved raspberry viruses to virus-free plants. **a** Virus acquisition time test: five large raspberry aphids (*Amphorophora idaei*) were placed on BRNV- or RYNV-infected raspberry leaves for 1 min, 5 min, 1 h, 5 h, or 24 h to find the optimal acquisition time; **b** Virus transmission test (Cryo): fifty aphids were allowed to feed for 1 h on BRNV- and RYNV-infected ‘Zlatá Královna’ (ZK) leaves excised from plants regenerated after cryopreservation. Aphids were then transferred to ten virus-free ‘Ninni’ plants and allowed to feed for 48 h. After two months, plants were tested by RT-PCR to check for virus status. A control group (Non-Cryo) was carried out by feeding aphids on leaves from non-cryopreserved ‘ZK’ plants and inoculating on five virus-free ‘Ninni’ plants. Created with BioRender.com

The aphid transmission experiment was performed as described by Sapkota et al. [3]. Adult wingless aphids from BRNV-free and RYNV-free colonies of large raspberry aphid (*Amphorophora idaei)* were used in the experiment. For the virus acquisition phase, aphids were allowed to feed on BRNV- and RYNV- infected leaves developed on cryopreserved virus-infected shoots for varying durations: 1 min, 5 min, 1 h, 5 h, or 24 h. Each acquisition group consisted of 5 aphids. After each feeding period, 5 aphids were immediately tested for virus acquisition to determine the optimal feeding time (Fig. 1a); while the internal control of cytochrome c oxidase subunit I (COI) was used [46]. To evaluate RYNV acquisition, an additional group of five aphids was allowed to feed for 1 h on RYNV-infected ‘LG’ plants. Aphids from this group were also tested by RT-PCR for RYNV presence with COI used as internal control.

To further evaluate transmission, 50 aphids were carefully placed on the upper surfaces of BRNV- and RYNV-infected raspberry plants of cryopreserved ‘ZK’ for 1 h to allow virus acquisition. These aphids were then transferred to 10 virus-free ‘Ninni’ plants and allowed to feed for 48 h before being removed. The inoculated plants were grown under greenhouse conditions for 2 months and subsequently tested for virus presence. A control treatment was established using non-cryopreserved BRNV- and RYNV-infected raspberry ‘ZK’ plants to feed the aphids. These aphids were then used to inoculate only 5 virus-free “Ninni” plants, ensuring a parallel comparison with the cryopreserved treatment. The detailed experiment process is shown in Fig. 1b.

### Experimental design and data analysis

For the experiment assessing the effect of shoot tip size on cryopreservation efficiency and virus preservation, each treatment consisted of three replicates, with 10 shoot tips per replicate (total 30 shoot tips per treatment). Survival rate was recorded two weeks after cryopreservation and defined as the percentage of shoot tips exhibiting green coloration. Regrowth rate was assessed eight weeks post-cryopreservation and defined as the percentage of shoot tips that developed into morphologically normal shoots (≥ 5 mm in length, with at least two fully expanded leaves). Statistical significance in survival and regrowth rates was analyzed using Student’s *t*-test (*P* < 0.05).

For virus preservation assessment, 10 regenerated shoots were randomly selected from each treatment for RT-PCR virus detection. The virus preservation rate was calculated as: (Number of virus-positive shoots after cryopreservation / 10) × (Total number of regenerated shoots after cryopreservation / 30) × 100%.

For the experiment assessing virus preservation across different raspberry cultivars, each treatment consisted of three replicates, with 10 shoot tips per replicate (total 30 shoot tips per cultivar). After cryopreservation and regeneration, 10 regenerated plants per cultivar were randomly selected for virus detection (as described above). The entire experiment was repeated twice, and the final virus preservation rate was calculated as the average of the two independent trails.

For virus localization, 10 randomly selected post-thaw shoot tips were subjected to ISH to examine viral distribution within the tissue.

## Results

### Virus detection by RT-PCR

RT-PCR was used to determine the virus status of the raspberry cultivars included in this study (Table 2). The results revealed variability in virus presence among the cultivars. ‘ZK’ tested positive for RBDV, BRNV, and RYNV. ‘FV’ were positive only for RYNV, while ‘TUM’ tested positive for RBDV and BRNV. ‘LG’ was infected with RYNV, ‘Bliss’ was positive for RBDV, and “MJ” tested positive for BRNV. In contrast, ‘Ninni’ remained virus-free, testing negative for RBDV, BRNV, and RYNV. ‘Stiora’ was first detected negative for RBDV, BRNV, and RYNV, however after up to 6 months of tissue culture, we have detected RYNV again in some tissue culture plants.

**Table 2:** Overview of virus infection status for eight raspberry cultivars used in this study

Cultivar	Growth Conditions	RBDV		BRNV	RYNV
Zlatá Královna (ZK)	In vitro	+		+	+
Tulameen (TUM)	In vitro	+		+	–
Fair View (FV)	In vitro	–		–	+
Stiora	In vitro	–		–	+
Ninni	in vitro (healthy)	–		–	–
Lloyd George (LG)	Greenhouse-cultivated (positive control)	–		–	+
Bliss	Greenhouse-cultivated (positive control)	+		–	–
Malling Jewel (MJ)	Greenhouse-cultivated (positive control)	–		+	–

Virus presence (+) or absence (–) was determined by reverse transcription polymerase chain reaction (RT-PCR) targeting raspberry bushy dwarf virus (RBDV), black raspberry necrosis virus (BRNV), and Rubus yellow net virus (RYNV). All plants were maintained under the listed growth conditions (in vitro or greenhouse) for a minimum of six months prior to testing

### Virus localization by in situ hybridization

**Fig. 2:**
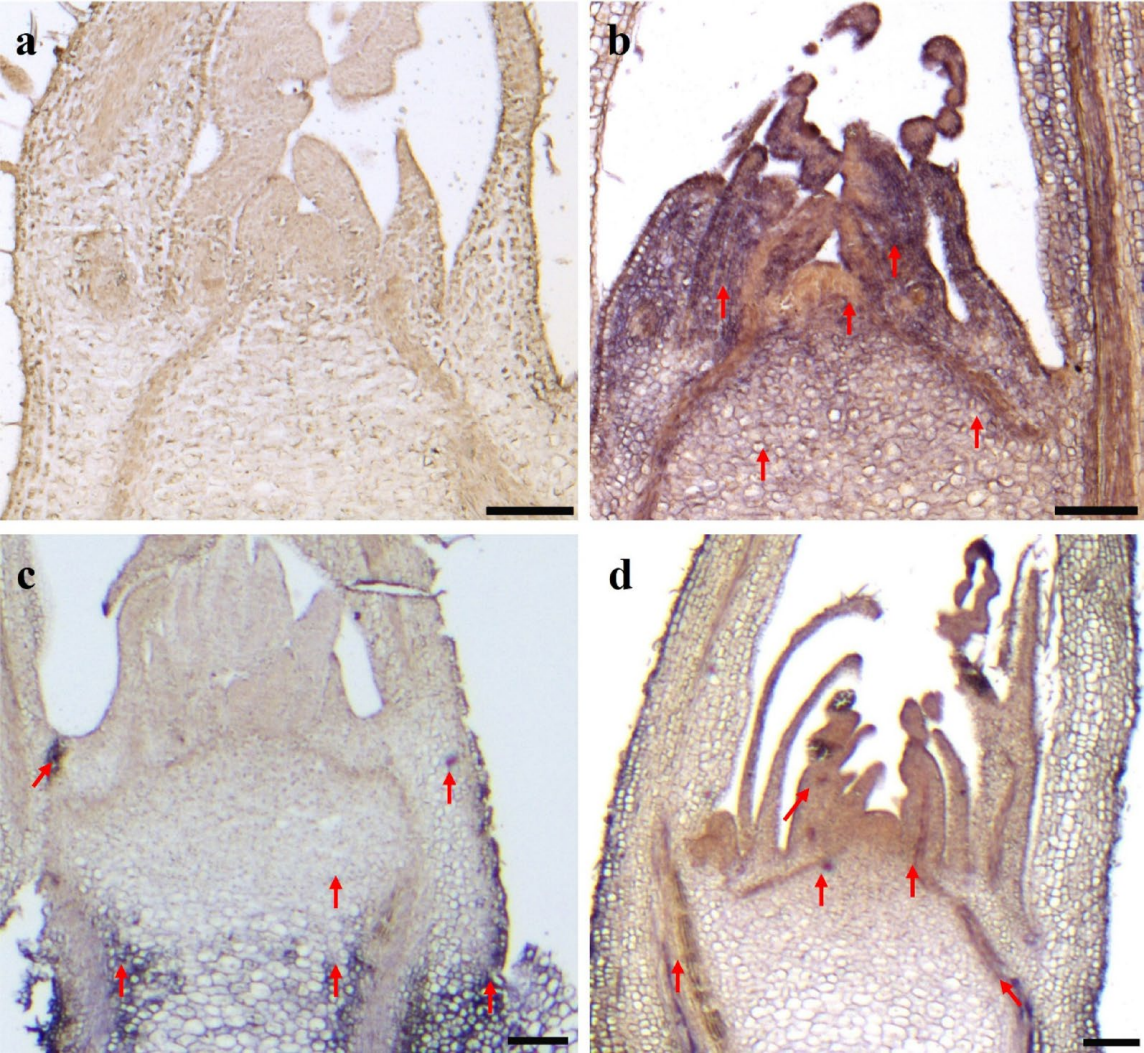
In situ hybridization showing localization of RBDV, BRNV, and RYNV in infected shoot tips of raspberry cultivars ‘Bliss’, ‘Malling Jewel (MJ)’, and ‘Lloyd George (LG)’. **a** Shoot apical meristem (SAM) of negative control with no probe. **b** SAM of ‘Bliss’ infected with RBDV. **c** SAM of ‘MJ’ infected with BRNV. **d** SAM of ‘LG’ infected with RYNV. Virus-infected cells show a purple-blue color reaction, as indicated by red arrows. Scale bars: 100 µm

ISH using strand-specific DIG-labeled antisense probes for RBDV, BRNV, and RYNV resulted in purple-blue color reactions in the shoot tips of raspberry cultivars ‘Bliss’, ‘MJ’, and ‘LG’, respectively (Fig. 2). No color reaction was observed when no probe was applied, no matter which cultivar mentioned above were used (Fig. 2a). In the RBDV-infected shoots of ‘Bliss’, a strong purple-blue color reaction was observed across the whole shoot tip, including the youngest LPs and AD (Fig. 2b). In the BRNV-infected shoots of ‘MJ’, a strong purple color reaction was distributed in older LPs, however, no color reaction was detected in youngest LPs and AD (Fig. 2c). In the RYNV-infected shoots of ‘LG’, RYNV was observed across whole shoot tip, including the youngest LPs (Fig. 2d).

**Fig. 3:**
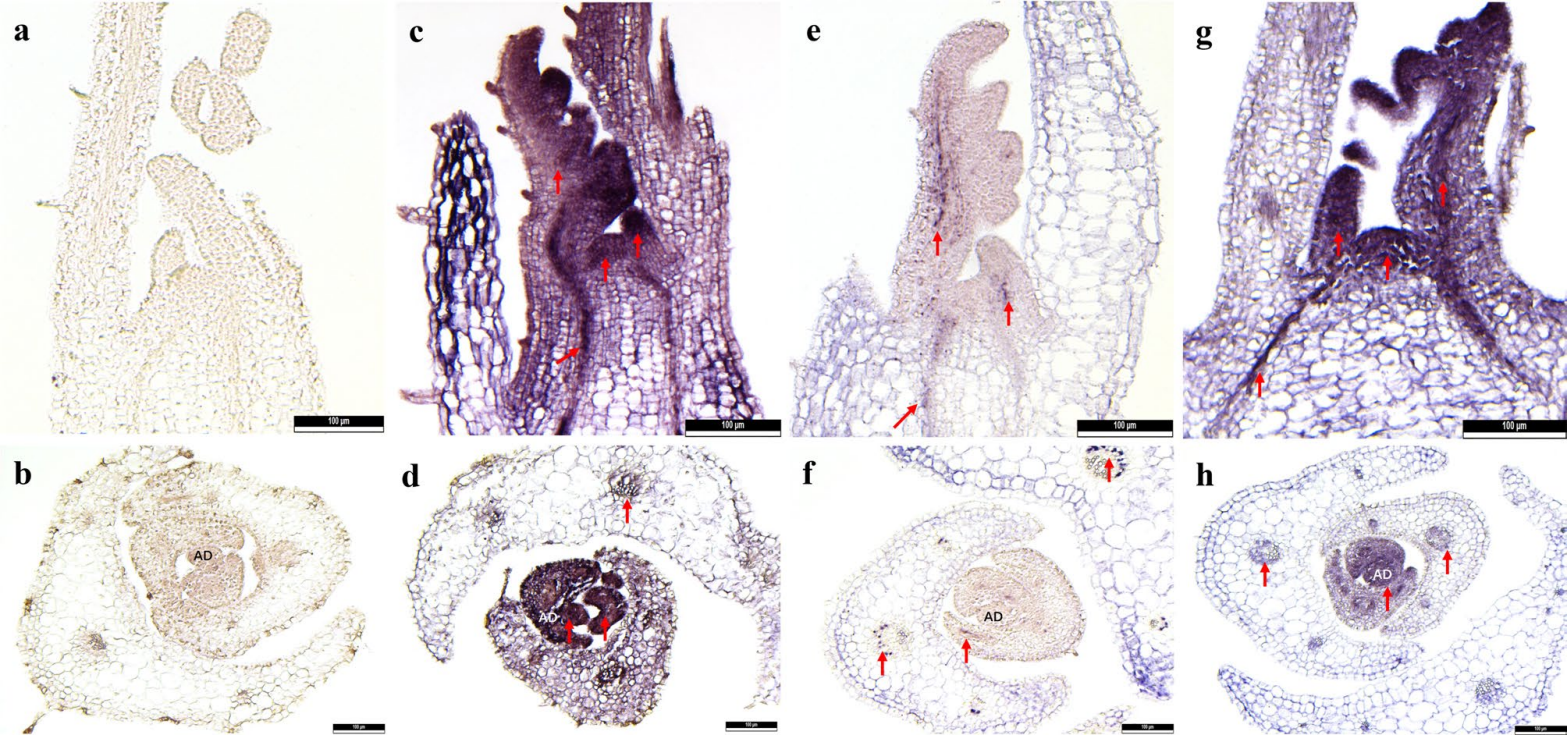
In situ hybridization showing localization of RBDV, BRNV and RYNV in infected shoot tips of raspberry cultivars ‘Zlatá Královna’. **a** Longitudinal sections of healthy shoot tip of virus-free ‘Ninni’ serve as negative control. **b** Cross section of healthy shoot tip of virus-free ‘Ninni’ serves as negative control. **c** Longitudinal section of RBDV-infected shoot tip. **d** Cross section of RBDV-infected shoot tips. **e** Longitudinal section of BRNV-infected shoot tip. **f** Cross section of RBNV-infected shoot tip. **g** Longitudinal section of RYNV-infected shoot tip. **h** Cross section of RYNV-infected shoot tip. Virus-infected cells show a purple-blue color reaction, as indicated by red arrows. AD = apical dome in **b**, **d**, **f**, and **h**. Scale bars = 100 μm

The distribution of RBDV, BRNV, and RYNV in infected shoot tips of raspberry ‘ZK’ is shown in Fig. 3 using both longitudinal and cross sections. No hybridization signal was observed in shoot tips of virus-free ‘Ninni’ used as the negative control (Fig. 3a and b). Strong purple-blue color reactions indicating viral localization were observed throughout the shoot tips for RBDV and RYNV localization, including the youngest LPs and AD (Fig. 3c, d, g, and h). In contrast, BRNV localization was detected in the older LPs, with no detectable signal in the youngest the AD (Fig. 3e and f).

### The effect of shoot tip size on shoot survival, regrowth, and virus preservation after cryopreservation

In the optimization experiment for shoot tip size, shoot tips measuring 1 mm showed the highest survival (93%) and regrowth (83%) rates compared to those of 0.5 mm and 2 mm in the raspberry cultivar ‘ZK’ under 20 min PVS2 exposure (Table 3). The raspberry cultivar ‘TUM’ also achieved the highest survival and regrowth rates with 1 mm shoot tips (94% and 87%, respectively) (Table 3). Similarly, the highest virus preservation efficiency, RBDV (83%, 87%), BRNV (66%, 52%), RYNV (83%, not tested) were achieved in 1 mm size shoot tips from the cryopreserved shoots of ‘ZK’ and ‘TUM’, respectively, as detected by RT-PCR 3 months after cryopreservation (Table 3).

**Table 3:** Effect of shoot tip size on survival, regrowth, and virus preservation following droplet-vitrification cryopreservation of raspberry cultivars ‘Zlatá Královna’ (ZK) and ‘Tulameen’ (TUM)

Raspberry cultivars	Shoot tip size (mm)	Survival (%)	Regrowth (%)	Virus preservation *(%)		
				RBDV	BRNV	RYNV
Zlatá Královna (ZK)	0.5	80b	47b	47	37	42
	1	93a	83a	83	66	83
	2	81ab	78a	70	63	78
Tulameen (TUM)	0.5	55c	38b	38	19	Not tested
	1	94a	87a	87	52	Not tested
	2	78b	69a	57	14	Not tested

* Virus preservation rate was calculated as: (number of virus-positive shoots after cryopreservation / 10) × (total number of regenerated shoots after cryopreservation/30) × 100%

Survival and regrowth rates were recorded at 2- and 8-weeks post-thaw, respectively. Virus preservation was assessed three months after cryopreservation by reverse transcription polymerase chain reaction (RT-PCR) for raspberry bushy dwarf virus (RBDV), black raspberry necrosis virus (BRNV), and Rubus yellow net virus (RYNV) in regenerated shoots

### The virus preservation of different raspberry cultivars

**Fig. 4:**
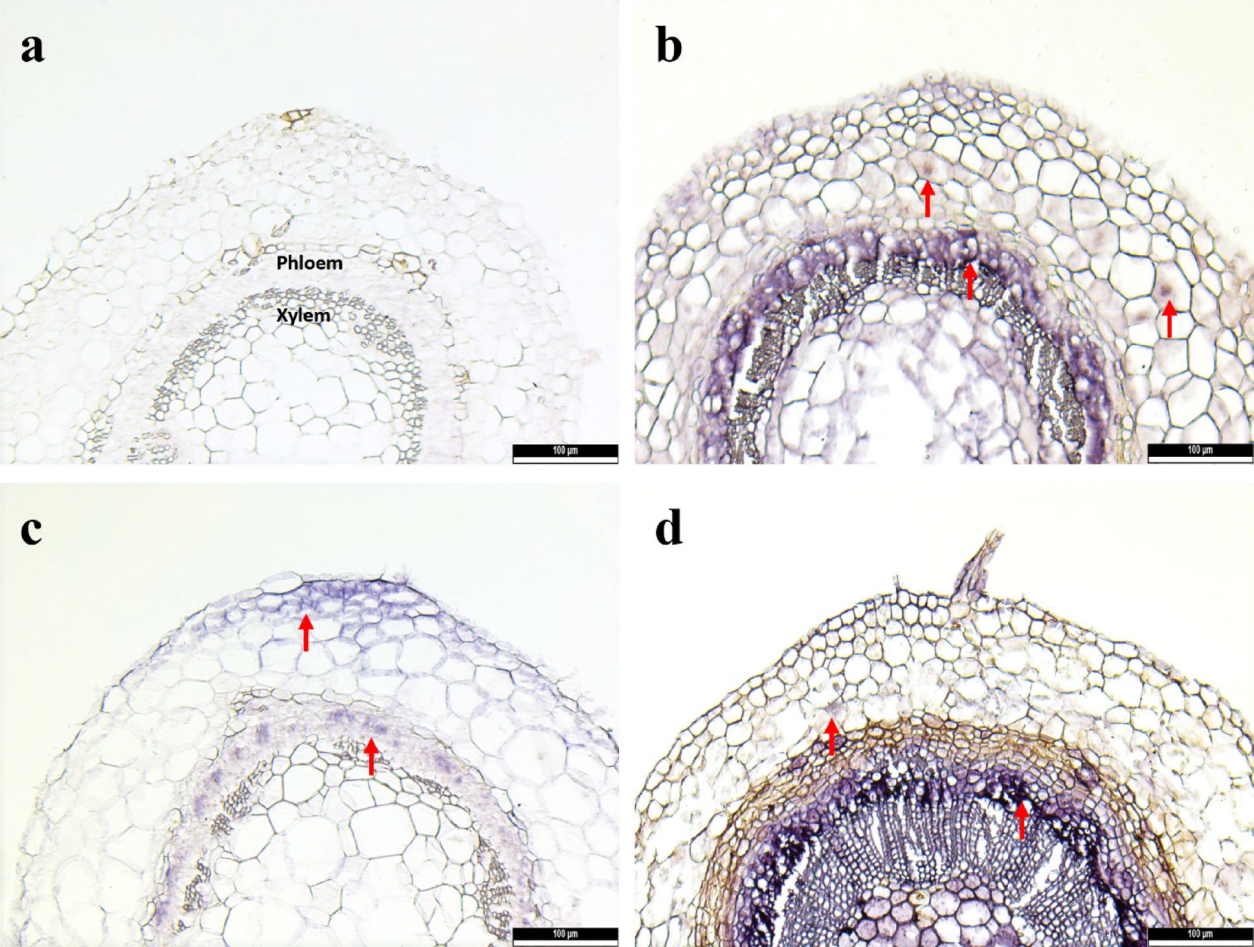
In situ hybridization showing localization of cryopreserved RBDV, BRNV and RYNV in cross-sections of infected shoot tips of raspberry cultivar ‘Zlatá Královna’. **a** Negative control of virus-free ‘Ninni’. **b** Localization of RBDV. **c** Localization of BRNV. **d** Localization of RYNV. Virus-infected cells show a purple-blue color reaction, as indicated by red arrows. Scale bars = 100 μm

Shoot tips (1 mm in length) from different raspberry cultivars were used for the cryopreservation of RBDV, BRNV, and RYNV, and the presence of the viruses was detected post-cryopreservation by RT-PCR and ISH. Preservation rates of 86% and 87% of RBDV were achieved in cultivars ‘ZK’ and ‘TUM’, respectively, while 86%, 96% and 94% virus preservation rate of RYNV was observed in the cultivars ‘ZK’, ‘FV’ and ‘Stiora’, respectively. Meanwhile, 66% and 45% of BRNV were preserved in cultivars ‘ZK’ and ‘TUM’, respectively (Table 4). Additionally, the viruses were tested by ISH in the stems of cultivar ‘ZK’ following cryopreservation. RBDV, BRNV, and RYNV were observed as a purple-blue color reaction in the phloem of the cross-section of stems (Fig. 4).

**Table 4:** Virus preservation across different raspberry cultivars. Detection of raspberry bushy Dwarf virus (RBDV), black raspberry necrosis virus (BRNV), and rubus yellow net virus (RYNV) by reverse transcription polymerase chain reaction (RT-PCR) in regenerated plants three months after cryopreservation

Raspberry cultivars	Virus cryopreservation* (%)		
	RBDV	BRNV	RYNV
Zlatá Královna (ZK)	86	66	86
Fair View (FV)	Not tested	Not tested	96
Stiora	Not tested	Not tested	94
Tulameen (TUM)	87	45	Not tested

* Virus preservation rate was the average of two independent trails, for each trail, virus preservation rate = (number of virus-positive shoots after cryopreservation / 10) × (total number of regenerated shoots after cryopreservation / 30) × 100%

### Virus activity verification by micrografting

The RBDV and BRNV activity was assessed through micrografting, using cryopreserved ‘ZK’ shoots as scions (Fig. 4a, f) and RBDV- and BRNV-free shoots of ‘Stiora’ as rootstocks (Fig. 4b, g). A faint purple color reaction was observed at 3 days post-micrografting, indicating the presence of RBDV and BRNV (Fig. 4c, h). After 7 and 14 days of post-micrografting, the purple color reaction had intensified considerably (Fig. 4d, i, e, j). The activity of RYNV was tested using virus-free ‘Ninni’ shoots as scions (Fig. 4k) and grafted onto cryopreserved RYNV-infected ‘ZK’ shoots as rootstocks (Fig. 4i). A strong purple color reaction was observed consistently at 3, 7, and 14 days post-micrografting (Fig. 4m, n, o). These results confirm that RBDV, BRNV, and RYNV remained active and were successfully transmitted from infected shoots to healthy shoots through micrografting.

**Fig. 5:**
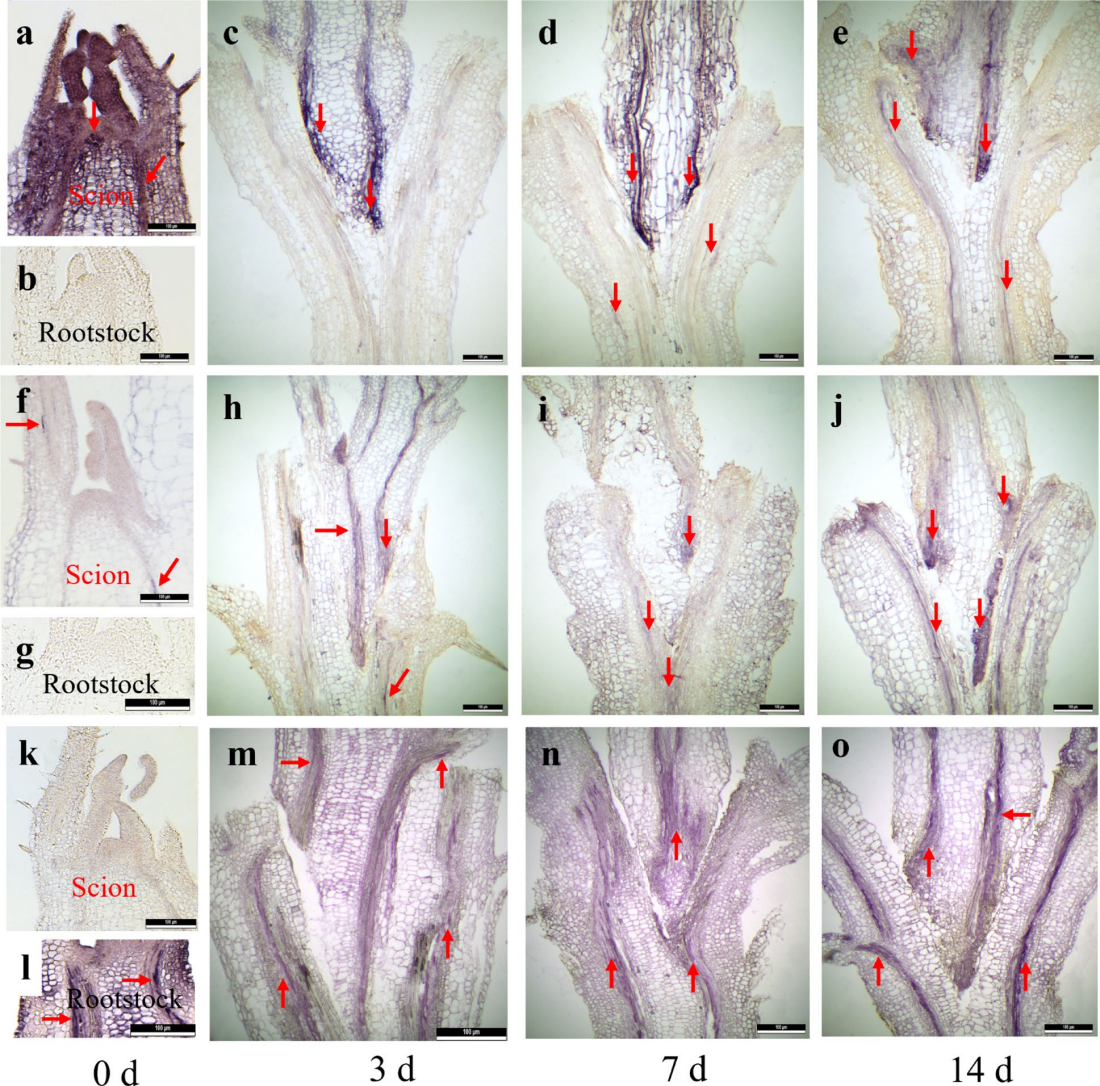
In situ hybridization showing localization of RBDV, BRNV, and RYNV in longitudinal sections of in vitro micrografted shoots. The micrografting combinations included: scions (cryopreserved shoots of ‘Zlatá Královna (ZK)’) grafted onto rootstocks (RBDV- and BRNV-free shoots of ‘Stiora’) for RBDV and BRNV transmission, and scions (virus-free shoots of ‘Ninni’) grafted onto rootstocks (cryopreserved shoots of ‘ZK’) for RYNV transmission. **a** Localization of RBDV in cryopreserved shoots of ‘ZK.’ **b** Localization of RBDV in shoots of ‘Stiora.’ **c**–**e** Localization of RBDV in micrografted shoots at days 3, 7, and 14, respectively. **f** Localization of BRNV in cryopreserved shoots of ‘ZK.’ **g** Localization of BRNV in shoots of ‘Stiora.’ **h**–**j** Localization of BRNV in micrografted shoots at days 3, 7, and 14, respectively. **k** Localization of RYNV in shoots of ‘Ninni.’ **l** Localization of RYNV in cryopreserved shoots of ‘ZK.’ **m**–**o** Localization of RYNV in micrografted shoots at days 3, 7, and 14, respectively. Virus-infected cells show a purple-blue color reaction, as indicated by red arrows. Scale bars = 100 μm

### Virus activity verification by aphids

Large raspberry aphid colonies (*Amphorophora idaei*) were initially screened for BRNV and RYNV using RT-PCR and confirmed to be free of both BRNV and RYNV. When aphids were fed on BRNV- and RYNV-infected raspberry plants of ‘ZK’ for 1 min, 5 min, 1 h, 5 h, and 24 h, BRNV was consistently acquired by the aphids and detected across all acquisition periods. In contrast, RYNV was not detected in any of the aphids following feeding on RYNV-infected ‘ZK’ plants. To verify aphids’ ability to acquire and transmit RYNV, aphids were fed on RYNV-infected plants of ‘LG’ (RYNV positive plants grown in greenhouse, not used for cryopreservation study) for 1 h, and RYNV was successfully detected in the aphids (Table 5).

**Table 5:** Detection of BRNV and RYNV in aphids (*Amphorophora idaei*) following acquisition feeding on infected raspberry plants at different time intervals

Cultivar	Acquisition time	BRNV	RYNV	COI
Zlatá Královna (ZK)	1 min	+	-	+
	5 min	+	-	+
	1 h	+	-	+
	5 h	+	-	+
	24 h	+	-	+
Lloyd George (LG)	1 h	Not tested	+	+

‘+’ = virus detected; ‘-’ = virus not detected. Aphids were allowed to feed on black raspberry necrosis virus (BRNV) or Rubus yellow net virus (RYNV) infected raspberry plants for different acquisition periods (1 min to 24 h). Virus presence was assessed in individual aphids using reverse transcription polymerase chain reaction (RT-PCR). The cytochrome c oxidase subunit I (COI) gene was used as an internal control to confirm RNA quality and RT-PCR validity

Virus infectivity post cryopreservation was evaluated through aphid-mediated transmission. Aphids were allowed to feed for 1 h on regenerated ‘ZK’ plants following cryopreservation (virus source) and then transferred to virus-free ‘Ninni’ plants for a 48- hour inoculation period. Two months after post-inoculation, RT-PCR analysis revealed BRNV presence in 3 out of 10 (30%) recipient ‘Ninni’ plants. In the control treatment, where aphids fed on non-cryopreserved ‘ZK’ plants, BRNV was detected in 1 out of 5 (20%) recipient plants (Table 6).   No RYNV was detected in any recipient ‘Ninni’ plants (Table 6).

**Table 6:** Detection of black raspberry necrosis virus (BRNV) and Rubus yellow net virus (RYNV) in recipient raspberry plants following aphid-mediated transmission

Virus donor plants	Recipient plants	BRNV	RYNV
Non-Cryo-ZK	Ninni 1	-	-
	Ninni 2	-	-
	Ninni 3	-	-
	Ninni 4	-	-
	Ninni 5	+	-
Cryo-ZK	Ninni 6	+	-
	Ninni 7	+	-
	Ninni 8	-	-
	Ninni 9	-	-
	Ninni 10	-	-
	Ninni 11	-	-
	Ninni 12	-	-
	Ninni 13	-	-
	Ninni 14	-	-
	Ninni 15	+	-

The table presents the virus detection results by reverse transcription polymerase chain reaction (RT-PCR) in virus-free ‘Ninni’ plants two months after aphid inoculation. Aphids had previously feed with the viruses through 1-hour feeding on donor plants, including both cryopreserved (Cryo-ZK) and non-cryopreserved (Non-Cryo-ZK) raspberry ‘Zlatá Královna’ plants

‘+’ = virus detected; ‘-’ = virus not detected

## Discussion and conclusion

The success of virus cryopreservation depends on several key factors, including the distribution of the virus within the preserved samples, the regeneration rate of shoot from cryopreserved shoot tips, and the subsequent proliferation of virus-infected shoots derived from these shoot tips [14, 29]. These factors play a critical role in evaluating the effectiveness of virus cryopreservation protocols. It is important to emphasize that cryopreservation does not eradicate viruses capable of infecting meristematic cells in the AD. Examples of such viruses include RBDV [47], Chrysanthemum stunt viroid (CSVd) [42], Pelargonium line pattern virus (PLPV) [48], apple hammerhead viroid (AHVd) [21] and ASGV [20]. In this study, we investigated the distribution of RBDV and RYNV in the shoot tips of cultivar ‘ZK’. A strong purple color reaction was observed in AD (Fig. 5d, h), confirming the localization of both viruses, and 86% cryopreservation frequencies of RBDV and RYNV were achieved using the droplet-vitrification method (Table 4). As mentioned in the introduction, the cryopreservation process enables the survival of cells located in the uppermost layers of the AD and within the youngest LPs, while other cells in the shoot tips are often damaged or die during the process [31–33]. Our results further confirm that viruses that can infect the AD, such as RBDV and RYNV, were effectively preserved using cryopreservation. These findings provide additional evidence supporting the compatibility of RYNV distribution and successful cryopreservation, thereby advancing our understanding of the preservation of viruses infecting meristematic tissues.

In the preceding discourse, it has become evident that the distribution of viruses significantly influences their cryopreservation. However, not every virus can infect the AD; for example, the apple stem pitting virus (ASPV) [49], and cucumber mosaic virus (CMV) [50]. This presents a particular challenge in the cryopreservation of these specific viruses. In this study, we investigated the cryopreservation of viruses that cannot infected the AD. Using ISH, BRNV was found to be distributed in the older LPs of the cultivar ‘MJ’ (Fig. 2c) and in the youngest LPs of the cultivar ‘ZK’ (Fig. 5e, f). A cryopreservation frequency of 66% was achieved for BRNV in cultivar ‘ZK’ using droplet-vitrification, while only 45% of BRNV was preserved in cultivar ‘TUM’ (Table 4). These results demonstrate that virus cryopreservation efficiency is strongly correlated with the distribution of the virus within the shoot tip. Viruses located far from the AD tend to have lower preservation rates, likely due to the limited survival of non-meristematic cells during the cryopreservation process. Additionally, the distribution patterns of BRNV and RYNV varied across different cultivars. For instance, the same virus showed distinct localization in different raspberry cultivars, providing greater opportunities for optimizing cryopreservation strategies. Such variability in virus distribution among cultivars highlights the importance of genotype-specific approaches to improve cryopreservation efficiency and supports the need for further research into the interplay between virus localization and preservation success.

It has been recognized that when cells of shoot tips are exposed to cryopreservation, those from the AD and youngest LPs are more likely to survive. This is due to their small vacuole size and low free-water content [13, 14, 19, 51]. There is a relationship where the higher the regeneration rate of cryopreservation, the higher the virus preservation rate. In other words, virus preservation is inversely proportional to virus eradication [29]. Li et al. (2018) reported that the size of shoot tips significantly affected shoot regrowth levels in cryopreserved shoot tips [19]. Larger shoot tips (1.5 mm) produced significantly higher shoot regrowth levels (52–60%) compared to smaller ones (0.5 mm, 30–38%). For PLRV, cryopreservation of 0.5-mm shoot tips did not produce any virus-preserved shoots, while 1.5-mm shoot tips resulted in the production of 35% of virus-preserved shoots. In this study, RBDV, BRNV, and RYNV could be preserved regardless of the size (0.5, 1, and 2 mm) of the shoot tips. However, 1 mm shoot tips achieved the highest virus preservation rates: 83% for RBDV and RYNV, and 66% for BRNV in cultivar ‘ZK’. Similarly, in cultivar ‘TUM’, 1 mm shoot tips achieved the highest rates, 87% for RBDV and 52% for BRNV (Table 3).

Another important factor influencing virus preservation during cryopreservation is the duration of exposure to PVS2. The length of PVS2 treatment impacts both the survival of host tissues and the stability of the virus. In this study, a 20-minute exposure to PVS2 was selected based on preliminary experiments aimed at optimizing shoot tip viability and regeneration [16, 28]. Previous studies, including work on potato virus eradication, have shown that longer PVS2 exposures can significantly affect virus survival and host recovery outcomes [52]. Prolonged exposure may enhance virus elimination by increasing cellular dehydration and damage to virus-infected tissues, whereas shorter durations may better preserve virus integrity. Therefore, optimizing PVS2 exposure is critical to achieve the objective, whether it is to eradicate viruses for production of virus-free plants or to preserve virus-infected tissues for research purposes. The balance between cryoprotection and virus viability must be carefully calibrated for each host-virus system.

Similar to the preservation of plant germplasm, genetic stability is a critical factor to consider, when preserving viruses using various methods. Once cryopreserved, cellular division and metabolic processes cease in the stored explant, enabling samples to be preserved indefinitely and thus minimizing changes to their genetic integrity [53]. Previous studies have reported that potato virus Y and watermelon mosaic virus retained infectivity after 22 and 32 months in LN, respectively, with no observed decline in transmission efficiency [39]. These findings support the resilience of purified plant viruses under prolonged cryostorage and underscore the need for future studies to evaluate similar long-term effects on virus-infected tissues [20, 31, 39, 54, 55]. Towill and Bonnart (2003) have reported that a cooling rate of 130 °C/s was calculated during the cryopreservation of shoot tips [56], indicating that a 2-minute treatment is sufficient for the shoot tips to reach the LN temperature and complete the process effectively. In the present study, RBDV, BRNV, and RYNV were detected using RT-PCR and ISH after 3 months of shoot regeneration, confirming the presence of these viruses in shoots after cryopreservation. Additionally, the viral activity of RBDV and BRNV was further verified by micrografting, successfully transferring the virus from infected shoots to healthy shoots.

BRNV and RYNV can be transmitted by aphids [25]. In this study, aphid transmission was used to assess the biological activity of the viruses after cryopreservation. BRNV from cryopreserved ‘ZK’ plants was successfully transmitted to healthy plants by aphid, confirming that the virus remained infective post-cryopreservation. In contrast, no transmission of RYNV was observed from cryopreserved ‘ZK’. Interestingly, RYNV in ‘LG’ plants, was successfully transmitted by aphids (Table 5). This observation is consistent with previous reports; Diaz-Lara et al. (2015) reported that the RYNV-BS strain lacks aphid transmissibility [57]. Based on our findings and previous research, we hypothesize that differences among RYNV strains may account for the observed disparities in aphid transmissibility.

In addition, we observed another interesting phenomenon about RYNV: with successive rounds of micropropagation for up to six months, originally virus-free shoots of ‘Stiora’ appeared to become RYNV-positive again. Previous findings have confirmed that RYNV can be integrated into red raspberry plants and even after being eradicated through thermotherapy and meristem-tip culture, RYNV can still be detected as positive by RT-PCR [57–60]. HO et al. (2024) conducted an analysis and validation of endogenous RYNV (endoRYNV) integrated into the genome of the red raspberry and determined that endoRYNV could be reactivated [61]. Meanwhile, genome sequencing of different raspberry cultivars found that the endoRYNV sequences may be limited to red raspberry. Our finding of RYNV re-detection in ‘Stiora’ after tissue culture can be due to this complexity of RYNV integration. Although RYNV is not known to reactivate into an infectious form like endogenous banana streak virus (eBSV) in *Musa* spp., the parallels highlight a broader issue: tissue culture stress can influence the expression or detection of integrated viral sequences [62–65]. Since RT-PCR cannot distinguish between episomal and integrated forms, additional molecular tools such as Southern blotting, rolling circle amplification, or long-read sequencing would be necessary to determine whether the detected RYNV is episomal, transcriptionally active, or a remnant of endoRYNV.

In conclusion, the raspberry viruses RBDV, BRNV, and RYNV can be successfully preserved in infected shoot tips using droplet-vitrification. Following cryopreservation, all three viruses remained detectable by RT-PCR and ISH after 3 months of shoot regeneration. The biological activity of BRNV was confirmed through both micrografting and aphid transmission, while RBDV and RYNV were verified only by micrografting. These results support shoot tip cryopreservation as an effective method for storage of biologically active raspberry viruses.

## Data Availability

No datasets were generated or analysed during the current study.
